# Exclusive breastfeeding among women with polycystic ovary syndrome versus women from a population-based cohort: a cohort study

**DOI:** 10.1186/s13006-026-00843-8

**Published:** 2026-04-18

**Authors:** Anne Engtrø Husby, Melanie Rae Simpson, Rebecka Dalbye, Tone Shetelig Løvvik, Eszter Vanky

**Affiliations:** 1 Department of Clinical and Molecular Medicine, Faculty of Medicine and Health Sciences, Norwegian University of Science and Technology, Trondheim, Norway; 2 Department of Obstetrics and Gynaecology, St Olav’s Hospital, Trondheim University Hospital, Postbox 3250 Torgarden, Trondheim, 7006 Norway; 3 Department of Public Health and Nursing, Faculty of Medicine and Health Sciences, Norwegian University of Science and Technology, Trondheim, Norway; 4 Department of Obstetrics and Gynaecology, Østfold Hospital Trust, Grålum, Norway; 5 Department of Nursing and Health Promotion, Faculty of Health Sciences, Oslo Metropolitan University, Oslo, Norway

**Keywords:** PCOS, Breastfeeding, BMI, Metformin, GDM, MoBa

## Abstract

**Background:**

Women with polycystic ovary syndrome (PCOS) face breastfeeding challenges. We aimed to compare exclusive breastfeeding rates in women with PCOS to those in a population-based cohort, and to explore the effect of metformin and metabolic factors on exclusive breastfeeding in women with PCOS.

**Methods:**

This cohort study included 653 women from the pilot, PregMet, and PregMet2 randomized trials on metformin in pregnant women with PCOS (2000–2017), and 63,927 women from the Norwegian Mother, Father and Child Cohort Study (MoBa) (1999–2008) as the reference group. Logistic regression estimated odds ratios for exclusive breastfeeding one month postpartum in women with PCOS versus MoBa women, and in women with PCOS randomized to metformin or placebo.

**Results:**

Women with PCOS reported less exclusive breastfeeding than MoBa women one month postpartum (adjusted OR (aOR) 0.52 (0.42–0.63)). Among women of normal weight, those with PCOS breastfed to the same extent as MoBa women (aOR 0.98 (0.63–1.54)), but less in the overweight or obesity categories (aOR 0.56 (0.39–0.82) and aOR 0.55 (0.40–0.76), respectively). However, differences across weight categories were not statistically significant (*p*-value for interaction = 0.088). We found no difference in exclusive breastfeeding between metformin and placebo-treated women with PCOS. Hypertensive disorders and gestational diabetes did not affect exclusive breastfeeding rate in PCOS.

**Conclusions:**

Exclusive breastfeeding did not differ between normal weight women with PCOS and women from the MoBa study. Among women with high BMI, women with PCOS breastfed less exclusively than MoBa women. In PCOS, neither metformin treatment, hypertension nor gestational diabetes affected exclusive breastfeeding.

**Supplementary Information:**

The online version contains supplementary material available at 10.1186/s13006-026-00843-8.

## Introduction

Polycystic ovary syndrome (PCOS) is the most common endocrine disorder in women [1]. Women with PCOS suffer more often from metabolic, psychological and reproductive comorbidities compared to women without PCOS [2], and run a higher risk for experiencing pregnancy complications such as gestational diabetes (GDM), hypertensive disorders and preterm birth [3–5].

In the Nordic countries, breastfeeding is the norm and 9–12 months paid parental leave gives women the opportunity to breastfeed [6]. In Norway, 98% of the mothers initiate breastfeeding, and most hospitals and public health services work in accordance with the ten steps of the baby-friendly hospital initiative [6–8].

Previous research suggests that women with PCOS breastfeed less than women without PCOS [9–14]. The possible mechanisms for impaired lactation in women with PCOS include hormonal influences of androgens, reduced progesterone levels, suboptimal prolactin or insulin dynamics, breast tissue hypoplasia, physical challenges related to comorbidities such as diabetes, obesity, or psychosocial factors [13–16]. Epidemiological studies with self-reported PCOS-diagnosis have found that women with PCOS breastfeed to the same extent as other women when adjusting for Body Mass Index (BMI), implying that obesity rather than PCOS-status per se has a negative influence on breastfeeding [10, 15, 17].

A case-control study found a significantly poorer metabolic health profile in women with poor breastmilk production [18]. In women with PCOS, we have previously shown that breast size increment during pregnancy has been associated with more breastfeeding [19], and high prolactin levels and breast size increment have been associated with more favourable metabolic health including lower BMI [16]. This suggests that women with PCOS and good metabolic health breastfeed better than those with poorer metabolic health, however the evidence is still scarce.

Metformin is predominantly used in type 2 diabetes to reduce insulin resistance [20]. It has no known teratogenic effects, yet it may affect the long-term metabolic health of the offspring [21]. Anecdotal evidence suggests that metformin may enhance milk supply [13], however, a pilot trial of metformin to enhance milk production following a diagnosis of insufficient milk supply found that metformin was unlikely to result in any clinically meaningful change in milk production [22]. An earlier study from our group on breastfeeding in women with PCOS with fewer participants found no evidence of beneficial effect of metformin use during pregnancy [11].

### Aim of the study

The main aim of this study was to investigate if exclusive breastfeeding rates differ between women with and without a clinically well-defined PCOS diagnosis. Secondary aims were to examine, in a larger sample, whether metformin has any impact on exclusive breastfeeding in women with PCOS, and to explore possible associations between exclusive breastfeeding and metabolic factors in women with PCOS.

## Methods and materials

### Design and participants

**Fig. 1:**
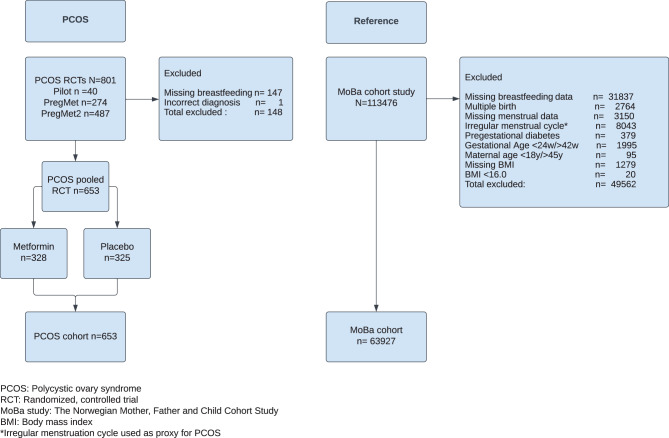
Flowchart of study inclusion: PCOS randomized controlled trials and MoBa cohort study. Inclusion criteria for all four studies were: available data on breastfeeding and BMI, maternal age between 18 and 45 years, singleton pregnancy of 24–42 weeks gestation and no previous diabetes. For the reference group we excluded participants with irregular menstruation or missing data on menstruation

The present cohort study consists of data from three randomized controlled trials of women with PCOS, the pilot, PregMet and PregMet2 studies, and a large population-based cohort study, the Norwegian Mother, Father and Child Cohort Study (MoBa) (see Fig. 1). In the randomized trials, pregnant women with PCOS were treated with metformin or placebo from first trimester and throughout the pregnancy. PCOS-diagnosis followed the Rotterdam criteria which includes two out of three following criteria: oligo/anovulation, hyperandrogenism and polycystic ovaries [23–25]. The pilot study [23] (2000–2003) included 40 women and found no effect of metformin compared to placebo on androgen levels in women with PCOS but suggested a possible reduction in pregnancy complications with metformin treatment. The PregMet study [24] (2005–2009) included 274 women and found no effect of metformin on pregnancy complications in women with PCOS. The PregMet2 study [25] (2012–2017) included 487 women from 14 hospitals in Norway, Sweden and Iceland, and found a reduction in preterm births and late miscarriages in women with PCOS treated with metformin. The study medicine metformin, the inclusion and exclusion criteria as well as the randomization processes were similar and are described in detail in the original studies [23–25].

The Norwegian Mother, Father and Child Cohort Study (MoBa) [26–28] is a population-based pregnancy cohort study conducted by the Norwegian Institute of Public Health. Participants were recruited from all over Norway from 1999 to 2008. The participation rate was 41%. The cohort includes approximately 114.500 children, 95.200 mothers and 75.200 fathers. The current study is based on version 12 of the quality-assured data files released for research in 2024. In this study, participants from the MoBa cohort were used as references.

### Patient involvement

A representative from the Norwegian PCOS interest organization has reviewed and provided feedback on the analyses for the present study. In the original studies, however, no patient representative was involved, as this was not required or generally practiced when the studies were planned and run.

### Ethical considerations

The Pilot, PregMet and PregMet2 studies and the MoBa study were approved by the appropriate Regional Committees for Health Research Ethics in Norway, Sweden and Iceland. The PregMet and PregMet2 studies were, in addition, registered at clinicaltrials.gov. All participants gave written consent. More details follow the main text of the article.

### Data collection

Women with missing breastfeeding data were excluded from all four studies. In the pilot, PregMet and PregMet2 studies, data on demographics, maternal anthropometry, obstetric and medical history were collected at inclusion, and the participants had follow-up visits in gestational weeks 19, 24 (PregMet) or 28 (PregMet2), 32 and 36. Data from birth and postpartum were collected from medical records and telephone interviews, and recorded in a Case Report Form either on paper or online (Web CRF version 2) [23–25]. In all, 653 women answered questions about breastfeeding after two months or during the first year after birth in the three studies on PCOS in pregnancy [11, 19, 25]. In the MoBa study, self-reported data on demographics, obstetric and medical history as well as anthropometry were gathered via questionnaires in pregnancy weeks 15 and 30. The MoBa women answered a breastfeeding questionnaire 6 months postpartum [28]. We excluded women with a possible PCOS-diagnosis from the reference (MoBa) population. As a proxy for PCOS, we used information about irregular menstruation (cycles less than 21 days or more than 35 days) or amenorrhea (>3 months with no menstrual period the last year before pregnancy). This would exclude roughly 85% of the women with PCOS in the MoBa population since approximately 85% of women with PCOS has oligo/amenhorrea [29]. To ensure the reference population was comparable to the PCOS-studies, we also excluded participants in the MoBa study on the following criteria: missing BMI or BMI < 16 kg/m^2^, multiple pregnancies, type 1 diabetes and manifest type 2 diabetes, women <18 years and >45 years old or pregnancies <24 weeks or >42 weeks. A total of 63,927 mother/child dyads were included from the MoBa study (Fig. 1).

### Statistical analysis

SPSS version 29 was used for the statistical analyses. Continuous variables are presented as means and standard deviations, and categorical variables as numbers and percentages. We compared baseline characteristics of participants in the pilot, PregMet and PregMet2 studies and the MoBa study, using t-test for continuous variables, and Chi-Square test, Likelihood ratio or Fischer’s exact test as appropriate for categorical variables.

Logistic regression was used to investigate the study aims. We present odds ratios (ORs) with 95% confidence intervals (CIs) for both univariate and multivariate analyses. For each aim, two models were run using the “enter” method in SPSS: one unadjusted and one adjusted model. Exploratory analyses were conducted to examine differences between PCOS and MoBa women within each BMI-category, including an interaction term between PCOS and BMI-category. The main results are presented in Table 3, and the secondary aims are addressed in Tables 4 and 5. *P*-values < 0.05 were considered statistically significant. No correction for multiple testing was deemed necessary.

The outcome variable breastfeeding was dichotomized into “exclusive breastfeeding” meaning the infant received only breastmilk and “partial or no breastfeeding”, ensuring harmonisation for the variable between the different primary studies. Possible confounding factors for each variable of interest were identified from literature, included in Directed acyclic graphs [30] (Fig. S1) and discussed in the research group. Ultimately, pregestational BMI, maternal age, parity, education and tobacco (smoking and snuff) were adjusted for. Since the relationship between BMI score and the log odds of breastfeeding was not linear, pregestational BMI was categorized as: normal weight (BMI 18.5–24.9 kg/m^2^), overweight (BMI 25.0–29.9 kg/m^2^) and obese (BMI ≥ 30 kg/m^2^). Since only two participants in the PCOS-group were underweight, including them in the further analyses was not meaningful. The dichotomous variable hypertensive disorder includes any hypertensive disorder in pregnancy including chronic hypertension, gestational hypertension, preeclampsia and HELLP syndrome (Haemolysis, elevated liver enzymes, low platelets). Information about triglycerides and HDL (High density lipoprotein) in first trimester was available for 231 women from the PregMet2 study.

Each mother-child dyad counted as one unit. In total, 3% (*n* = 20) of women in the PCOS group and 13% (*n* = 8464) in the MoBa cohort participated more than once due to separate pregnancies. To check for the effect of this possible dependency between observations, a sensitivity analysis was done where all women participating during more than one pregnancy were excluded. This did not alter the results (Table S1).

## Results

Table 1 shows that women with PCOS were more often primiparous, had higher BMI, and more often gave birth by caesarean section compared with the reference (MoBa) population, and their offspring had a lower average birthweight. Age, education, civil status as well as proportion of preterm births were comparable between the groups (Table 1). Baseline characteristics of women with PCOS treated with metformin and placebo were comparable, except for BMI being higher in the metformin group (Table 2).

**Table 1:** Baseline data and birth characteristics for women with PCOS (pilot/PregMet/PregMet2 pooled) and reference women (MoBa)

	PCOS (n = 653)	Reference (n = 63927)	p-value*
**Age (years at inclusion) Mean (SD)**	29.7 (4.37)	29.9 (4.46)	0.26
**Civil Status n (%)**			
Married/co-habitant	444 (97.8)^199^	61903 (97.3)^282^	0.66
Other	10 (2.2)	1742 (2.7)	
**Education n (%)**			
Elementary School	18 (4.0)^199^	3562 (5.8)^2770^	0.16
High School	127 (28.0)	15943 (26.2)	
College/University	309 (68.1)	41397 (68.0)	
**BMI (kg/m**^**2)**^ **Mean (SD)**	28.36 (6.313)	24.44 (4.093)	<0.001
**BMI (kg/m**^**2**^**) WHO categories**			
BMI < 18.5 (Underweight)	2 (0.3)	935 (1.5)	<0.001
BMI 18.50–24.99 (Normal weight)	228 (34.9)	40577 (63.5)	
BMI 25.00–29.99 (Overweight)	182 (27.9)	16219 (25.4)	
BMI 30.00–34.99 (Obesity class 1)	150 (23.0)	4647 (7.3)	
BMI 35.00–39.99 (Obesity class 2)	63 (9.6)	1198 (1.9)	
BMI > 40.00 (Obesity class 3)	28 (4.3)	351 (0.5)	
**Tobacco n (%)**	39 (6.0) [1]	4506 (8.0)^7388^	<0.001
**Parity**			
Para 0	372 (57.0)	30314 (47.4)	<0.001
Para1 or more	281 (43.0)	33613 (52.6)	
**Mode of delivery n (%)**			
Vaginal birth	528 (80.9)	51350 (86.4)^4521^	<0.001
Caesarean section	125 (19.1)	8056 (13.6)	
**Preterm birth (24.0–36.6) n (%)**	37 (5.7)	2812 (4.8)^5901^	0.33
**Gestational age Mean (SD)**	39.7 (1.82)	39.7 (1.87)^5901^	0.97
**Birth weight offspring Mean (SD)**	3538 (558)	3592 (546)^4578^	0.01

Numbers in n (%)^m^ or Mean (SD)^m^ as appropriate where m is number missing in column

Statistical analyses performed with independent samples t-test for continuous data and Pearson’s chi-squared test, Likelihood ratio or Fisher’s exact test as appropriate for categorical data

*Significance level 0.05

BMI: Body mass index

PCOS: Polycystic ovary syndrome

MoBa: The Norwegian Mother, Father and Child Cohort Study

**Table 2:** Baseline data and birth characteristics for women with PCOS (pilot/PregMet/PregMet2 pooled) metformin vs placebo

	Metformin (n = 328)	Placebo (n = 325)	p-value*
**Age (years at inclusion) Mean (SD)**	29.8 (4.53)	29.6 (4.20)	0.57
**Civil Status n (%)**			
Married/co-habitant	222 (98.2)^102^	222 (97.4)^97^	0.75
Other	4 (1.8)	6 (2.6)	
**Education n (%)**			
Elementary School	9 (4.0)^102^	9 (3.9)^97^	0.60
High School	68 (30.1)	59 (25.9)	
College/University	149 (65.9)	160 (70.2)	
**BMI (kg/m**^**2)**^ **Mean (SD)**	28.9 (6.32)	27.8 (6.26)	0.02
**BMI (kg/m**^**2**^**) WHO-categories n (%)**			
BMI < 18.5 Underweight)	0 (0)	2 (0.6)	0.01
BMI 18.50–24.99 (Normal weight)	105 (32.0)	123 (37.8)	
BMI 25.00–29.99 (Overweight)	80 (24.4)	102 (31.4)	
BMI 30.00–34.99 (Obesity class 1)	88 (26.8)	62 (19.1)	
BMI 35.00–39.99 (Obesity class 2)	38 (11.6)	25 (7.7)	
BMI > 40.00 (Obesity class 3)	17 (5.2)	11 (3.4)	
**Tobacco n (%)**	21 (6.4) [1]	18 (5.5)	0.74
**Medical history n (%)**			
Eating disorder	14 (6.7)^119^	14 (6.7)^116^	1.00
Hypertension	14 (4.5)^16^	10 (3.3)^20^	0.44
Depression	56 (18.0)^17^	45 (14.7)^19^	0.27
Migraine	47 (15.1)^17^	52 (17.0)^19^	0.52
Asthma	24 (7.7)^15^	36 (11.7)^18^	0.09
**Parity n (%)**			
Para 0	193 (58.8)	179 (55.1)	0.33
Para1 or more	135 (41.2)	146 (44.9)	
**Mode of delivery n (%)**			
Vaginal birth	264 (80.5)	264 (81.2)	0.81
Caesarean section	64 (19.5)	61 (18.8)	
**Transfer NICU n (%)**	25 (12.0)^119^	23 (11.0)^116^	0.76
**Preterm birth (24.0–36.6) n (%)**	11 (3.4)	26 (8.0)	0.01
**Gestational age Mean (SD)**	39.7 (1.68)	39.6 (1.95)	0.36
**Birth weight offspring Mean (SD)**	3552 (533.6)	3524 (582.6)	0.54

Numbers in n (%)^m^ or Mean (SD)^m^ as appropriate where m is number missing in column

Statistical analyses performed with independent samples t-test for continuous data and Pearson’s chi-squared test, Likelihood ratio or Fisher’s exact test as appropriate for categorical data

*Significance level 0.05

BMI: Body mass index

NICU: Newborn intensive care unit

PCOS: Polycystic ovary syndrome

Fewer women with PCOS breastfed exclusively at one month postpartum compared with the MoBa women (aOR 0.52, 95% CI 0.42–0.63) (Table 3).

**Table 3:** Exclusive breastfeeding one month postpartum in women with PCOS (pilot/PregMet/PregMet2 pooled) vs reference women (MoBa)

		PCOS (n = 651)	MoBa (n = 62992)	Univariate		Multivariate	
		n (%)	n (%)	OR (95% CI)	p-value	aOR (95% CI)	p-value*
BMI ≥ 18.5	Exclusive breastfeeding	483 (74.0)	52772 (83.8)	0.55 (0.46–0.66)	<0.001	0.52 (0.42–0.63)	<0.001
	Partial or no breastfeeding	170 (26.0)	10220 (16.2)				
BMI 18.5–24.9	Exclusive breastfeeding	200 (87.7)	34981 (86.4)	1.13 (0.76–1.67)	0.56	0.98 (0.63–1.54)	0.94
(Normal weight)	Partial or no breastfeeding	28 (12.3)	5513 (13.6)				
BMI 25.0–29.9	Exclusive breastfeeding	126 (69.6)	13181 (81.2)	0.54 (0.39–0.74)	<0.001	0.56 (0.39–0.82)	0.003
(Overweight)	Partial or no breastfeeding	55 (30.4)	3058 (18.8)				
BMI ≥ 30	Exclusive breastfeeding	155 (64.0)	4558 (73.6)	0.64 (0.49–0.83)	<0.001	0.55 (0.40–0.76)	<0.001
(Obesity)	Partial or no breastfeeding	87 (36.0)	1639 (26.4)				
Interaction term BMI*PCOS					0.014		0.088

Binary logistic regression

Multivariate analyses adjusted for age, parity, education and tobacco

*Significance level 0.05

BMI: Body mass index

CI: Confidence interval

MoBa: The Norwegian Mother, Father and ChildCohort Study

OR: Odds ratio

PCOS: Polycystic ovary syndrome

Analyses according to BMI-category, adjusted for maternal age, parity, tobacco use and educational level (Fig. S1), showed no difference in exclusive breastfeeding in normal weight women with PCOS compared to normal weight MoBa women (aOR 0.98, 95% CI 0.63–1.54) (Table 3). Women with PCOS compared with MoBa women had lower odds for exclusive breastfeeding in the overweight and obese BMI categories (aOR 0.56, 95% CI 0.39–0.82 and aOR 0.55, 95% CI 0.40–0.76, respectively), however the formal test for interaction between PCOS and BMI-category was not statistically significant for the adjusted model (*p*-value for interaction = 0.088). Increasing BMI was associated with progressively lower exclusive breastfeeding rates in the reference population (Table 3).

In the PCOS-group at one month postpartum, there was no difference in exclusive breastfeeding between those receiving metformin or placebo (OR 1.05, 95% CI 0.74–1.49) (Table 4).

**Table 4:** Effect of metformin on exclusive breastfeeding patterns of women with PCOS at one month postpartum

	Metformin (n = 328)	Placebo (n = 325)			
	n (%)	n (%)	OR	95% CI	p-value*
Exclusive breastfeeding	241 (73.5)	242 (74.5)	1.05	0.74–1.49	0.77
Partial or no breastfeeding	87 (26.5)	83 (25.5)			

Binary logistic regression. Breastfeeding data from 653 women from the pilot, PregMet and PregMet2 studies. Non-adjusted (randomized trial)

*Significance level 0.05

CI: Confidence interval

OR: Odds ratio

PCOS: Polycystic ovary syndrome

Among women with PCOS, those with overweight and obesity class 1 (BMI 30.0–34.9), 2 (BMI 35.0–39.9) and 3 (BMI 40 and above) had progressively lower odds for exclusive breastfeeding compared with those with normal weight, with the odds ratios ranging from OR 0.40 (95% CI 0.22–0.73) for overweight women to OR 0.16 (95% CI 0.05–0.48) for women with obesity class 3 (Table 5).

**Table 5:** Univariate and multivariate analyses of metabolic status in women with PCOS exclusively breastfeeding vs partially/not breastfeeding at one month post-partum

	Exclusive breastfeeding	Partial/no breastfeeding	Univariate			Multivariate		
	n (%)	n (%)	OR	95% CI	p-value*	OR	95% CI	p-value*
^1^BMI 18.5–24.99 (Reference)	200 (87.7)	28 (12.3)						
BMI 25–29.99 (Overweight)	126 (69.6)	55 (30.4)	0.32	0.20–0.54	<0.001	0.40	0.22–0.73	0.003
BMI 30.0–34.99 (Obesity class 1)	102 (68.0)	48 (32.0)	0.30	0.18–0.50	<0.001	0.34	0.18–0.62	>0.001
BMI 35–39.99 (Obesity class 2)	37 (58.7)	26 (41.3)	0.20	0.11–0.37	<0.001	0.22	0.10–0.49	>0.001
BMI 40+ (Obesity class 3)	16 (55.2)	13 (44.8)	0.16	0.07–0.38	<0.001	0.16	0.05–0.48	0.001
^2^Hypertensive disorders**	43 (63.2)	25 (36.8)	0.57	0.34–0.97	0.04	0.77	0.44–1.33	0.34
No hypertensive disorders**	438 (75.1)	145 (24.9)						
^2^GDM	128 (71.1)	52 (28.9)	0.82	0.56–1.21	0.32	0.99	0.67–1.49	0.98
No GDM	353 (74.9)	118 (25.1)						
^3^Triglycerides*** mean (SD)	1.13 (0.51)	1.48 (0.83)	0.44	0.27–0.71	<0.001	0.49	0.30–0.80	0.004
^2^HDL*** mean (SD)	1.62 (0.37)	1.50 (0.33)	2.74	1.10–6.87	0.03	2.35	0.91–6.02	0.08

Binary logistic regression. Breastfeeding data from 651 women from the pilot, PregMet and PregMet2 studies with BMI ≥ 18.5

^1^Adjusted for education, parity, age and tobacco, ^2^adjusted for BMI, ^3^adjusted for BMI and age in multivariate analysis

*Significance level 0.05

**Hypertensive disorders include chronic hypertension, gestational hypertension, preeclampsia and HELLP syndrome

***Analyses of 231 women from PregMet2

BMI: Body mass index

CI: Confidence interval

GDM: Gestational diabetes

HDL: High density lipoprotein

OR: Odds ratio

PCOS: Polycystic ovary syndrome

Hypertensive disorders in pregnancy and GDM were not significantly associated with exclusive breastfeeding in women with PCOS when adjusted for BMI. Adjusted for BMI and maternal age, higher triglyceride levels gave lower odds for exclusive breastfeeding (Table 5).

## Discussion

### Main findings

Fewer women with PCOS breastfed exclusively at one month postpartum compared with MoBa women. The negative association between PCOS and exclusive breastfeeding was greater and remained statistically significant in the subgroups of women with overweight or obesity. However, among those who had normal weight, the proportion of women with exclusive breastfeeding was approximately equal between women with PCOS and MoBa women. Although we observed stronger associations between PCOS and lower exclusive breastfeeding rates in the overweight and obese weight categories, compared to normal weight, the difference was not statistically significant on formal testing of the interaction.

### Interpretation

The present study confirms that, in general, women with PCOS breastfeed less exclusively than other women. This is in line with previous clinical observations and cohort studies [10, 12, 13, 17]. It is well documented that high pregestational BMI is associated with poorer breastfeeding [31, 32], and women with PCOS have, on average, higher BMI when entering pregnancy compared to women without PCOS [2]. Previous studies suggest that overweight and obesity account for the poorer breastfeeding among women with PCOS [15, 17]. We observe lower odds for exclusive breastfeeding with increasing BMI in both women with PCOS and in MoBa women. However, in the present study, we show that PCOS status may be an additional factor to high BMI that influences breastfeeding negatively. This novel insight is of importance to both women with PCOS as well as health care providers giving advice to women on breastfeeding. Midwives, lactation consultants, and other health care professionals should balance their advice to avoid adding to the burden of mothers who struggle or are not able to breastfeed [33, 34].

Birth by caesarean section, low mean birthweight, small for gestational age and transfer to neonatal intensive care are more common outcomes among neonates of mothers with PCOS [3], and also of mothers with high BMI [35]. These factors may be mediators between high BMI and breastfeeding.

The most common reason for ceasing breastfeeding is perceived low milk supply, and women report a desperate struggle to enhance their milk production [36, 37]. Women with PCOS suffer from poorer body image and lower self-esteem [38]. Struggling with breastfeeding may be interpreted as yet another failure [36, 39] and linked to feelings of shame or guilt [40]. Women call for more knowledge among health care professionals to inform and prepare them in advance of breastfeeding challenges [36]. Informed from this study, both women and health care personnel may be more prepared for the possibility of an alternative neonate feeding plan, should breastfeeding prove challenging.

This study not only contributes to new knowledge on PCOS, overweight and breastfeeding, but also nuances the picture. Despite differences among groups, most women with PCOS and overweight or obesity, breastfed exclusively at one month postpartum. This may reflect Nordic conditions with high level of education, generous parental leave schemes, and systematic breastfeeding support [6, 7], that attenuate socioeconomic imbalances.

It is worth noting that women with PCOS and normal weight seem to breastfeed just as well as the MoBa women, which is in line with previous reports [15, 17]. Thus, the prevention of overweight and obesity in young women is important not only for their long-term metabolic health but may also be important for their ability to breastfeed. The 2023 evidence-based guidelines for assessment and management of PCOS highlights lifelong healthy lifestyle and the prevention of excessive weight gain already from adolescence [1].

The present study supports the evidence that pregestational hormonal and/or metabolic disturbances such as PCOS, high BMI and elevated triglycerides are associated with less breastfeeding. A cross-sectional study on duration of lactation, maternal metabolic profile and body composition (the Norwegian EBBA I-study) [41] found that women who breastfed longer, seemed more metabolically healthy around 5 years after birth. However, pregestational metabolic health was not taken into consideration. A longitudinal cohort study concluded that pre-pregnant metabolic health affects breastfeeding as well as long-term metabolic health [42], suggesting that poor breastfeeding and poor long-term metabolic health may have a common pregestational cause. Other studies have concluded that breastfeeding protects neither the offspring against the effects of maternal pregestational BMI and excessive gestational weight gain [43] nor the mother against cardiometabolic risk factors [44]. Promoting breastfeeding based on its alleged benefit for both maternal and offspring long-term metabolic health may thus be biased and should be done with caution.

Metformin during pregnancy did not impact breastfeeding in women with PCOS, which confirmed the results from our smaller previous study [11]. We found no independent associations between GDM or hypertensive disorder on breastfeeding in women with PCOS. This contrasts to previous research on breastfeeding women in general, where a recent review suggests that GDM is associated with less breastfeeding [37]. Another review reveals that GDM and hypertensive disorders in pregnancy are risk factors for delayed lactogenesis II which is predictive for early cessation of breastfeeding [45].

### Strength and limitations

Strengths: This study includes a large, clinically well-defined group of women with PCOS and a large reference population. Combining data from intervention trials and a large cohort study provided a broader evidence base and enabled comparisons between groups, strengthening the robustness of our findings. Stratifying by BMI-category allowed comparisons not only between women with PCOS and MoBa women, but also to compare these two groups within the same BMI range. All studies had high response rates on breastfeeding; in the pilot, PregMet and PregMet2 81%, and in the MoBa-study 72% answered the questions on neonatal feeding [26, 28].

Limitations: Breastfeeding data was self-reported in all four studies [23–26], introducing potential recall and information bias due to differences in timing and data collection. However, long-term recall for breastfeeding has been validated [46]. Assessing exclusive breastfeeding at one timepoint only limits insights into duration, however those who successfully establish breastfeeding are likely to continue [6]. Lack of confirmed PCOS-diagnosis and use of irregular menstruation as a proxy for PCOS in the reference (MoBa) group may lead to misclassification by including some women with PCOS, potentially diluting observed associations. As participation was voluntary, the study population may not fully represent all women with PCOS or the general population. Finally, combining data from three RCTs and one cohort study introduces heterogeneity in participant selection and study design, which may affect internal validity through selection bias and external validity due to differences in population characteristics in the original studies. To reduce this, we applied similar inclusion/exclusion criteria as in the pilot, PregMet and PregMet2 studies when we selected the reference group (MoBa).

## Conclusion

In general, women with PCOS breastfed exclusively to a lesser extent than MoBa women at one month after birth. The rate of exclusive breastfeeding was similar in normal weight women with PCOS and normal weight women from the MoBa study. However, among women with overweight or obesity, the presence of PCOS may be an additional negative factor for exclusive breastfeeding. This new insight is of clinical importance both for women with PCOS and for health care personnel. Exclusive breastfeeding in women with PCOS was not impacted by metformin in pregnancy, hypertension or GDM. Future research should explore additional factors possibly influencing exclusive breastfeeding in women with PCOS, including weight trajectories during life, gestational weight gain, development of breast tissue and inflammation.

## Electronic supplementary material

Below is the link to the electronic supplementary material.

Supplementary Material 1

Supplementary Material 2

## Data Availability

Some or all datasets generated during and/or analysed during the current study are not publicly available but are available from the corresponding author on reasonable request. For questions related to data from the MoBa study, please contact the MoBa study administration at MoBaadm@fhi.no.

## Abbreviations

PCOS: Polycystic ovary syndrome
MoBa: The Norwegian Mother, Father and Child Cohort Study
BMI: Body Mass Index
GDM: Gestational diabetes

## References

[1] Teede H, et al. International evidence-based guideline for the assessment and management of polycystic ovary syndrome 2023. Monash University; 2023.

[2] Cooney LG, Dokras A. Beyond fertility: polycystic ovary syndrome and long-term health. Fertil Steril. 2018;110(5):794–809. 10.1016/j.fertnstert.2018.08.021. 30316414 10.1016/j.fertnstert.2018.08.021

[3] Bahri Khomami M, Hashemi S, Shorakae S, Harrison CL, Piltonen TT, Romualdi D, et al. Systematic review and meta-analysis of birth outcomes in women with polycystic ovary syndrome. Nat Commun. 2024;15(1):5592. 10.1038/s41467-024-49752-6. 38965241 10.1038/s41467-024-49752-6 PMC11224419

[4] Bahri Khomami M, Shorakae S, Hashemi S, Harrison CL, Piltonen TT, Romualdi D, et al. Systematic review and meta-analysis of pregnancy outcomes in women with polycystic ovary syndrome. Nat Commun. 2024;15(1):5591. 10.1038/s41467-024-49749-1. 38965226 10.1038/s41467-024-49749-1 PMC11224312

[5] Yu H-F, Chen H-S, Rao D-P, Gong J. Association between polycystic ovary syndrome and the risk of pregnancy complications: a PRISMA-compliant systematic review and meta-analysis. Med (Baltim). 2016;95(51):e4863. 10.1097/MD.0000000000004863. 10.1097/MD.0000000000004863 PMC5181798 28002314

[6] Theurich MA, Davanzo R, Busck-Rasmussen M, Díaz-Gómez NM, Brennan C, Kylberg E, et al. Breastfeeding rates and programs in Europe: a survey of 11 national breastfeeding committees and representatives. J Pediatr Gastroenterol Nutr. 2019;68(3):400–07. 10.1097/MPG.0000000000002234. 30562307 10.1097/MPG.0000000000002234

[7] Helleve A, Lande B. Amming og spedbarns kosthold. Landsomfattende undersøkelse 2013. Helsedirektoratet. 2014.

[8] Helsedirektoratet. Nasjonal faglig retningslinje for spedbarnsernæring [National guidelines for infant nutrition] 2016 [updated 08 July 2021]. Available from: https://www.helsedirektoratet.no/retningslinjer/spedbarnsernaering.

[9] Harrison CL, Teede HJ, Joham AE, Moran LJ. Breastfeeding and obesity in PCOS. Expert Rev Endocrinol Metab. 2016;11(6):449–54. 10.1080/17446651.2016.1239523. 30058915 10.1080/17446651.2016.1239523

[10] Joham AE, Nanayakkara N, Ranasinha S, Zoungas S, Boyle J, Harrison CL, et al. Obesity, polycystic ovary syndrome and breastfeeding: an observational study. Acta Obstet Gynecol Scand. 2016;95(4):458–66. 10.1111/aogs.12850. 26782709 10.1111/aogs.12850

[11] Vanky E, Isaksen H, Haase Moen M, Carlsen SM. Breastfeeding in polycystic ovary syndrome. Acta Obstet Gynecol Scand. 2008;87(5):531–35. 10.1080/00016340802007676. 18446536 10.1080/00016340802007676

[12] Marasco L, Marmet C, Shell E. Polycystic ovary syndrome: a connection to insufficient milk supply? J Hum Lact. 2000;16(2):143–48. 10.1177/089033440001600211. 11153345 10.1177/089033440001600211

[13] Marasco LA. Unsolved mysteries of the human Mammary Gland: defining and redefining the critical questions from the lactation Consultant’s perspective. J Mammary Gland Biol Neoplasia. 2014;19(3–4):271–88. 10.1007/s10911-015-9330-7. 26084427 10.1007/s10911-015-9330-7

[14] Kam RL, Cullinane M, Amir LH. Breast hypoplasia and polycystic ovary syndrome: is there a link? Clin Lactation. 2021;12(4):159–67.

[15] Rassie K, Mousa A, Joham A, Teede HJ. Metabolic conditions including obesity, diabetes, and polycystic ovary syndrome: implications for breastfeeding and breastmilk composition. Semin Reprod Med. 2021;39(03/04):111–32. 10.1055/s-0041-1732365. 34433215 10.1055/s-0041-1732365

[16] Underdal MO, Salvesen Ø, Schmedes A, Andersen MS, Vanky E. Prolactin and breast increase during pregnancy in PCOS: linked to long-term metabolic health? Eur J Endocrinol. 2019;180(6):373–80. 10.1530/EJE-19-0002. 30991360 10.1530/EJE-19-0002

[17] Rassie K, Dhungana RR, Mousa A, Teede H, Joham Anju A. Maternal metabolic conditions as predictors of breastfeeding outcomes: insights from an Australian cohort study. Acta Obstet Gynecol Scand. 2024;103(8):1570–83. 10.1111/aogs.14868. 38715284 10.1111/aogs.14868 PMC11266642

[18] Nommsen-Rivers LA, Wagner EA, Roznowski DM, Riddle SW, Ward LP, Thompson A. Measures of maternal metabolic health as predictors of severely low milk production. Breastfeed Med. 2022;17(7):566–76. 10.1089/bfm.2021.0292. 35475660 10.1089/bfm.2021.0292 PMC9299530

[19] Vanky E, Nordskar JJ, Leithe H, Hjorth-Hansen AK, Martinussen M, Carlsen SM. Breast size increment during pregnancy and breastfeeding in mothers with polycystic ovary syndrome: a follow-up study of a randomised controlled trial on metformin versus placebo. BJOG. 2012;119(11):1403–09. 10.1111/j.1471-0528.2012.03449.x. 22827167 10.1111/j.1471-0528.2012.03449.x

[20] Foretz M, Guigas B, Viollet B. Metformin: update on mechanisms of action and repurposing potential. Nat Rev Endocrinol. 2023;19(8):460–76. 10.1038/s41574-023-00833-4. 37130947 10.1038/s41574-023-00833-4 PMC10153049

[21] Hanem LGE, Stridsklev S, Júlíusson PB, Salvesen Ø, Roelants M, Carlsen SM, et al. Metformin use in PCOS pregnancies increases the risk of offspring overweight at 4 years of age: follow-up of two RCTs. J Clin Endocrinol Metab. 2018;103(4):1612–21. 29490031 10.1210/jc.2017-02419

[22] Nommsen-Rivers L, Thompson A, Riddle S, Ward L, Wagner E, King E. Feasibility and acceptability of metformin to augment low milk supply: a pilot randomized controlled trial. J Hum Lact. 2019;35(2):261–71. 10.1177/0890334418819465. 30629889 10.1177/0890334418819465 PMC8992687

[23] Vanky E, Salvesen KA, Heimstad R, Fougner KJ, Romundstad P, Carlsen SM. Metformin reduces pregnancy complications without affecting androgen levels in pregnant polycystic ovary syndrome women: results of a randomized study. Hum Reprod. 2004;19(8):1734–40. 15178665 10.1093/humrep/deh347

[24] Vanky E, Stridsklev S, Heimstad R, Romundstad P, Skogøy K, Kleggetveit O, et al. Metformin versus placebo from first trimester to delivery in polycystic ovary syndrome: a randomized, controlled multicenter study. J Clin Endocrinol Metab. 2010;95(12):E448–55. 20926533 10.1210/jc.2010-0853

[25] Løvvik TS, Carlsen SM, Salvesen Ø, Steffensen B, Bixo M, Gómez-Real F, et al. Use of metformin to treat pregnant women with polycystic ovary syndrome (PregMet2): a randomised, double-blind, placebo-controlled trial. Lancet Diabetes Endocrinol. 2019;7(4):256–66. 30792154 10.1016/S2213-8587(19)30002-6

[26] Magnus P, Birke C, Vejrup K, Haugan A, Alsaker E, Daltveit AK, et al. Cohort profile update: the Norwegian mother and child cohort study (MoBa). Int J Epidemiol. 2016;45(2):382–88. 27063603 10.1093/ije/dyw029

[27] Magnus P, Irgens LM, Haug K, Nystad W, Skjærven R, Stoltenberg C, et al. Cohort profile: the Norwegian mother and child cohort study (MoBa). Int J Epidemiol. 2006;35(5):1146–50. 10.1093/ije/dyl170. 16926217 10.1093/ije/dyl170

[28] NIPH. Norwegian mother, father and child cohort study (MoBa). 2024. Available from: https://www.fhi.no/moba-en.

[29] Azziz R, Carmina E, Dewailly D, Diamanti-Kandarakis E, Escobar-Morreale HF, Futterweit W, et al. The androgen excess and PCOS society criteria for the polycystic ovary syndrome: the complete task force report. Fertil Steril. 2009;91(2):456–88. 18950759 10.1016/j.fertnstert.2008.06.035

[30] Textor J, van der Zander B, Gilthorpe MS, Liśkiewicz M, Ellison GTH. Robust causal inference using directed acyclic graphs: the R package ‘dagitty’. Int J Epidemiol. 2017;45(6):1887–94. 10.1093/ije/dyw341. 10.1093/ije/dyw341 28089956

[31] Ren Z, Zhang A, Zhang J, Wang R, Xia H. Role of perinatal biological factors in delayed lactogenesis II among women with pre-pregnancy overweight and Obesity. Biol Res Nurs. 2022;24(4):459–71. 10.1177/10998004221097085. 35505584 10.1177/10998004221097085

[32] Jin X, Lai CT, Perrella SL, McEachran JL, Gridneva Z, Geddes DT. Maternal breast growth and Body mass index are associated with low milk production in women. Nutrients. 2024;16(17):2854. 10.3390/nu16172854. 39275171 10.3390/nu16172854 PMC11397153

[33] Smyth D, Hyde A. Discourses and critiques of breastfeeding and their implications for midwives and health professionals. Nurs Inq. 2020;27(3):e12339. 10.1111/nin.12339. 31919926 10.1111/nin.12339

[34] Rueda C, Bright MA, Roussos-Ross D, Montoya-Williams D. Exclusive breastfeeding promotion policies: whose oxygen mask are we prioritizing? J Perinatol. 2022;42(8):1141–45. 10.1038/s41372-022-01339-z. 35347245 10.1038/s41372-022-01339-z PMC8960073

[35] Vats H, Saxena R, Sachdeva MP, Walia GK, Gupta V. Impact of maternal pre-pregnancy body mass index on maternal, fetal and neonatal adverse outcomes in the worldwide populations: a systematic review and meta-analysis. Obes Res Clin Pract. 2021;15(6):536–45. 10.1016/j.orcp.2021.10.005. 34782256 10.1016/j.orcp.2021.10.005

[36] Kam RL, Bennetts SK, Cullinane M, Amir LH. “I didn’t want to let go of the dream”: exploring women’s personal stories of how their low milk supply was discovered. Sexual Reprod Healthcare. 2024;40:100953. 10.1016/j.srhc.2024.100953. 10.1016/j.srhc.2024.100953 38437771

[37] Jin X, Perrella SL, Lai CT, Taylor NL, Geddes DT. Causes of low milk supply: the roles of estrogens, progesterone, and related external factors. Adv Nutr. 2024;15(1):100129. 10.1016/j.advnut.2023.10.002. 37832920 10.1016/j.advnut.2023.10.002 PMC10831895

[38] Davitadze M, Malhotra K, Khalil H, Hebbar M, Tay CT, Mousa A, et al. Body image concerns in women with polycystic ovary syndrome: a systematic review and meta-analysis. Eur J Endocrinol. 2023;189(2):R1–9. 10.1093/ejendo/lvad110. 37619990 10.1093/ejendo/lvad110

[39] Bresnahan M, Zhuang J, Goldbort J, Bogdan-Lovis E, Park SY, Hitt R. Made to feel like less of a woman: the experience of stigma for mothers who do not breastfeed. Breastfeed Med. 2020;15(1):35–40. 10.1089/bfm.2019.0171. 31859523 10.1089/bfm.2019.0171

[40] Jackson L, De Pascalis L, Harrold J, Fallon V. Guilt, shame, and postpartum infant feeding outcomes: a systematic review. Matern Child Nutr. 2021;17(3):e13141. 10.1111/mcn.13141. 33491303 10.1111/mcn.13141 PMC8189225

[41] Tørris C, Thune I, Emaus A, Finstad SE, Bye A, Furberg A-S, et al. Duration of lactation, maternal metabolic profile, and body composition in the Norwegian EBBA I-study. Breastfeed Med. 2013;8(1):8–15. 10.1089/bfm.2012.0048. 23057641 10.1089/bfm.2012.0048

[42] Velle-Forbord V, Skråstad RB, Salvesen Ø, Kramer MS, Morken NH, Vanky E. Breastfeeding and long-term maternal metabolic health in the HUNT study: a longitudinal population-based cohort study. BJOG. 2019;126(4):526–34. 10.1111/1471-0528.15538. 30461169 10.1111/1471-0528.15538

[43] Ohlendorf JM, Robinson K, Garnier-Villarreal M. The impact of maternal BMI, gestational weight gain, and breastfeeding on early childhood weight: analysis of a statewide WIC dataset. Prev Med. 2019;118:210–15. 10.1016/j.ypmed.2018.11.001. 30412742 10.1016/j.ypmed.2018.11.001

[44] Øhman EA, Fossli M, Rasmussen KM, Winkvist A, Løland BF, Holven KB, et al. Effects of breastfeeding promotion intervention and dietary treatment in postpartum women with overweight and obesity: results from a randomized controlled trial on weight and cardiometabolic risk factors. J Nutr. 2024;154(8):2448–58. 10.1016/j.tjnut.2024.06.006. 38901636 10.1016/j.tjnut.2024.06.006

[45] Peng Y, Zhuang K, Huang Y. Incidence and factors influencing delayed onset of lactation: a systematic review and meta-analysis. Int Breastfeed J. 2024;19(1):59. 10.1186/s13006-024-00666-5. 39175092 10.1186/s13006-024-00666-5 PMC11342634

[46] Schneider BC, Cata-Preta BO, Gräf DD, Silva D, Santos FS, Dias MS, et al. Validation of maternal recall on exclusive breastfeeding 12 months after childbirth. Public Health Nutr. 2020;23(14):2494–500. 32456727 10.1017/S136898002000018X PMC10200606

