# Supplementary material for: Exclusive breastfeeding among women with polycystic ovary syndrome versus women from a population-based cohort: a cohort study
Source: Int Breastfeed J. 2026 Apr 18;21:54. doi: 10.1186/s13006-026-00843-8 (PMC13231535; doi:10.1186/s13006-026-00843-8)
Supplement: Supplementary file 1 — Supplementary Material 1 [file 13006_2026_843_MOESM1_ESM.pptx]

## Slide 1
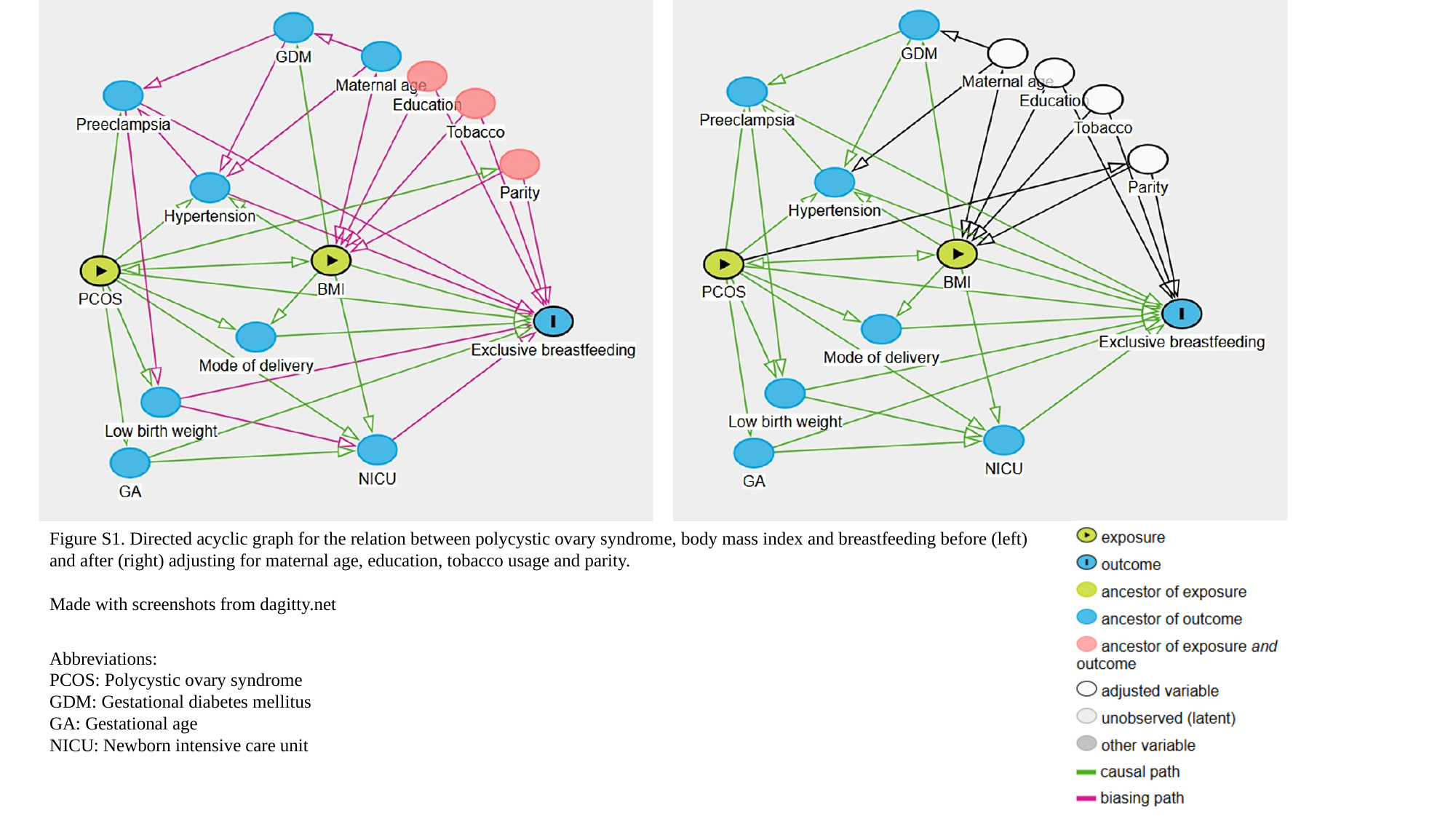

Figure S1. Directed acyclic graph for the relation between polycystic ovary syndrome, body mass index and breastfeeding before (left) and after (right) adjusting for maternal age, education, tobacco usage and parity.
Made with screenshots from dagitty.net
Abbreviations:
PCOS: Polycystic ovary syndrome
GDM: Gestational diabetes mellitus
GA: Gestational age
NICU: Newborn intensive care unit
