# Supplementary material for: Exclusive breastfeeding among women with polycystic ovary syndrome versus women from a population-based cohort: a cohort study
Source: Int Breastfeed J. 2026 Apr 18;21:54. doi: 10.1186/s13006-026-00843-8 (PMC13231535; doi:10.1186/s13006-026-00843-8)
Supplement: Supplementary file 2 — Supplementary Material 2 [file 13006_2026_843_MOESM2_ESM.docx]

**Table S1. Exclusive breastfeeding one month postpartum in women with PCOS (Pilot/PregMet/PregMet2 pooled) vs reference women (MoBa). Sensitivity analysis excluding double participation.**

|  |  | **PCOS (n=639)** | **MoBa (n=55340)** | |  |  |  |
| --- | --- | --- | --- | --- | --- | --- | --- |
|  |  | **n (%)** | **n (%)** | **OR (95% CI)** | **P-value** | **aOR (95% CI)** | **p-value*** |
| BMI ≥ 18.5 | Exclusive breastfeeding | 471 (73.7) | 46199 (83.5) | 0.55 (0.46-0.66) | < 0.001 | 0.52 (0.42-0.64) | < 0.001 |
|  | Partial or no breastfeeding | 168(26.3) | 9141 (16.5) |  |  |  |  |
| BMI 18.5-24.9 | Exclusive breastfeeding | 197 (87.9) | 30268 (86.1) | 1.18 (0.79-1.76) | 0.43 | 1.00 (0.64-1.56) | 0.99 |
| (Normal weight) | Partial or no breastfeeding | 27 (12.1) | 4881 (13.9) |  |  |  |  |
| BMI 25.0-29.9 | Exclusive breastfeeding | 125 (69.8) | 11750(81.0) | 0.54 (0.39-0.75) | < 0.001 | 0.57 (0.39-0.83) | 0.003 |
| (Overweight) | Partial or no breastfeeding | 55 (30.2) | 2749 (19.0) |  |  |  |  |
| BMI ≥ 30 | Exclusive breastfeeding | 149 (63.1) | 4181 (73.5) | 0.62 (0.47-0.81) | < 0.001 | 0.55 (0.40-0.76) | <0.001 |
| (Obesity) | Partial or no breastfeeding | 87 (36.9) | 1511 (26.5) |  |  |  |  |

Binary logistic regression.

Adjusted for age, parity, education and tobacco

*Significance level 0.05

BMI: Body mass index

CI: Confidence interval

MoBa: The Norwegian Mother, Father and Child Cohort Study

OR: Odds ratio

PCOS: Polycystic ovary syndrome
